# Docetaxel-Loaded Novel Nano-Platform for Synergistic Therapy of Non-Small Cell Lung Cancer

**DOI:** 10.3389/fphar.2022.832725

**Published:** 2022-03-02

**Authors:** Xing Feng, Xiaoling Xiong, Shenglin Ma

**Affiliations:** ^1^ Department of Thoracic Surgery, The Affiliated Hangzhou Hospital of Nanjing Medical University, Hangzhou, China; ^2^ Department of Thoracic Surgery, Affiliated Hangzhou First People’s Hospital, Zhejiang University School of Medicine, Hangzhou, China; ^3^ Department of Nephrology, Sir Run Run Shaw Hospital, Zhejiang University School of Medicine, Hangzhou, China; ^4^ Department of Oncology, The Affiliated Hangzhou Hospital of Nanjing Medical University, Hangzhou, China

**Keywords:** non-small cell lung cancer, targeted therapy, immunotherapy, hotothermal therapy, cGAS-STING

## Abstract

Nowadays, non-small cell lung cancer (NSCLC) is threatening the health of all mankind. Although many progresses on treatment of lung cancer have been achieved in the past few decades, the current treatment methods are still traditional surgery, radiotherapy, and chemotherapy, which had poor selectivity and side effects. Lower-toxicity and more efficient treatments are in sore need. In this paper, a smart nanodelivery platform based on photothermal therapy, chemotherapy, and immunotherapy was constructed. The nanoparticles are composed of novel photothermal agents, Mn-modified phthalocyanine derivative (Mn^III^PC), docetaxel (DTX), and an effective targeting molecule, hyaluronic acid. The nanoplatform could release Mn^2+^ from Mn^III^PC@DTX@PLGA@Mn^2+^@HA(MDPMH) and probably activate tumor immunity through cGAS-STING and chemotherapy, respectively. Furthermore, DTX could be released in the process for removal of tumor cells. The “one-for-all” nanomaterial may shed some light on treating NSCLC in multiple methods.

## Introduction

Nowadays, lung cancer is still one of the most fatal tumors worldwide and the main cause of cancer-related mortality (Lim, 2016). Lung cancer is classified into small cell lung cancer (SCLC) and non-small cell lung cancer (NSCLC) according to the different histological performance, in which NSCLC comprises about 85% of total cases (Jonna and Subramaniam, 2019; Lee and Cheah, 2019). The mainstay of treatments for NSCLC is still the conventional therapies, including chemotherapy, surgery, radiotherapy, and synergistic therapy. The physical damage caused by surgical treatment, dose dependence, low selectivity, and side effects of chemotherapy and radiotherapy often bring great suffering to the patient (Camidge et al., 2019; Duma et al., 2019). Therefore, it is urgent to develop a new strategy which is effective against NSCLC but low side effects to normal tissues.

Photothermal therapy (PTT), utilizing photothermal agents [e.g., noble metal nanoparticles (Liu et al., 2020; Zheng et al., 2020), transition metal sulfides (Ai et al., 2021; Dhas et al., 2021), black phosphorus (Jana et al., 2020), phthalocyanines (PCs) (Taratula et al., 2015; Jin et al., 2019; Li et al., 2019)], could efficiently absorb light and convert photon energy into heat and cause the thermal ablation of adjacent tumor cells (Camidge et al., 2019). However, some drawbacks, particularly poor thermal stability and low biocompatibility, had severely limited its wide application in biomedicine. Recently, metal phthalocyanines (MPCs) have caught the attention of researchers with strong NIR region absorption, high stability coefficient, and low phototoxicity. For example, Song et al. (2018) designed a smart theranostic nanoplatform based on a hyaluronic acid-doped polypyrrole-coated bismuth selenide loading with a zinc phthalocyanine nanodish for multimodal imaging-guided combined phototherapy, showing an excellent combined therapeutic effect. Moreover,the combination of PTT and immunotherapy could improve the scavenging capacity of tumor was testified (Li et al., 2017). For example, a photothermal agent IR820 and a programmed death-ligand 1 antibody were loaded into a lipid gel depot, which increased the recruitment of tumor-infiltrating lymphocytes and booted T-cell activity against tumors (Chen et al., 20198).

Further, it was reported that Mn^2+^ could be endocytosed by macrophages, dendritic cells, and lymphocytes and activates the innate immune response, which indicated that Mn^2+^ is a potential immunotherapy strategy (Huang et al., 2019). Hyaluronic acid (HA), composed of repeated units of β-4 linked D-glucuronic acid and β-3 linked N-acetyl-D-glucosamine, has been widely served in antitumor therapies because of its glorious biocompatibility and biodegradable character (Lv et al., 2020). Moreover, HA is the ligand for CD44 receptors, which are overexpressed in multiple tumors (Mattheolabakis et al., 2015; Salwowska et al., 2016).

Based on the current scientific studies, docetaxel (DTX) is one of the well-known and practically effective chemical anticancer agents for the inhibition of NSCLC both as a single agent and in combination treatments (Gao et al., 2019). Several effective drug delivery systems were designed to deliver DTX into tumor cells with high selectivity and efficacy while minimizing negative effects (Jing et al., 2018; Deng et al., 2020). Nanotechnology is a significant method which can be utilized to achieve cancer targeting and efficient treatment while avoiding unnecessary side effects (Rafiei and Haddadi, 2017).

**FIGURE 1:**
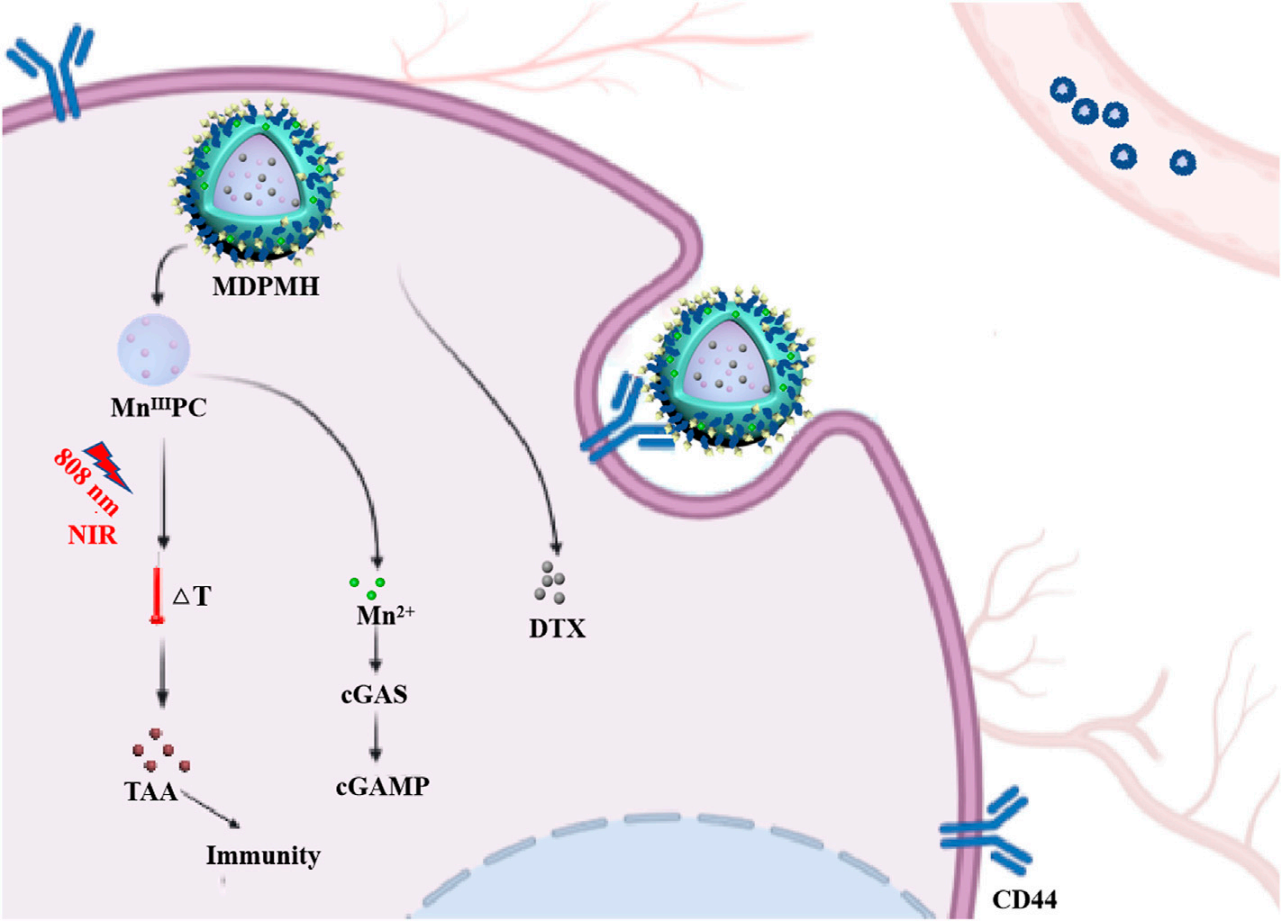
Schematic illustration of combinational therapy of PTT, chemotherapy, and immunotherapy (MDPMH) *via* a combined all-in-one and all-in-control strategy.

In this study, an intelligent nanoplatform combining PTT, chemotherapy, and immunotherapy was designed (Figure 1). Firstly, a novel sonosensitizer, an Mn-modified phthalocyanine derivative (Mn^III^PC), and an HA amphiphilic molecule (HA-HDA) were synthesized, respectively. Secondly, the DTX-loaded nanoparticles (Mn^III^PC@DTX@PLGA@Mn^2+^@HA, MDPMH) were prepared by mixing DTX, Mn^III^PC, HA-HDA, Mn^2+^, and PLGA under ultrasound. The physical and chemical properties and cytotoxicity to NSCLC cells of MDPMH were investigated subsequently. MDPMH nanoparticles could handily be internalized by cancer cells due to overexpressed CD44-mediated endocytosis. Mn^III^PC induced much heat in tumor cells under the irradiation of an 808-nm near-infrared laser; Mn^2+^ deposited on the surface of nanocarriers could activate tumor immunity through cGAS-STING. What is more, DTX released from the carrier could inhibit the proliferation of tumor cells with the biodegradation of HA and nanocarriers. This strategy may shed some light on treating NSCLC in multiple methods.

## Experimental Section

### Materials

1,4,8,11,15,18,22,25-Octabutoxy-29H,31H-phthalocyanine [(BuO)_8_PCH_2_], 1,3-diphenylisobenzofuran (DPBF), and polylactic-co-glycolic acid (PLGA, 50:50) were purchased from Sigma-Aldrich (Shanghai, China). N-Hydroxysuccinimide (NHS), N-(3-dimethylaminopropyl)-N′-ethyl carbodiimide hydrochloride (EDC·HCl), and DTX were obtained from Sangon Biotech (Shanghai) Co., Ltd. HA (10 kDa) was bought from Rhawn Science and Technology (Shanghai, China) Co., Ltd. Manganese chloride (MnCl_2_), 1-hexadecanamine (HDA), and hyaluronidase (HAase) were bought from Shanghai Yuanye Bio-Technology Co., Ltd. Dimethyl sulfoxide (DMSO), dimethylformamide (DMF), dichloromethane (DCM), ethanol, and chloroform were derived from Solarbio (Beijing, China). All reagents were of analytical grade and had no additional purification.

### Cell Lines

A549 cells were cultured in RPMI 1640 media supplemented with 10% fetal bovine serum and 1% penicillin–streptomycin solution under a 5% CO_2_ atmosphere at 37°C.

### The Synthesis of Mn(III) (BuO)_8_PCH_2_ (Mn^III^PC)

(BuO)_8_PCH_2_ (109.3 mg, 1.0 mmol, 1.0 eq) and MnCl_2_ (0.5 mg, 4.0 mmol, 4.0 eq) were dropped into 4 ml DMF under nitrogen atmosphere for 10 min. The above mixture was maintained at 180°C for 3 min. When the reaction system was cooled to room temperature spontaneously, it was dropped into 30 ml water with stirring. The mixture was centrifuged at 12,000 rpm for 5 min and washed with water for two times. The crude product was purified by silica gel column chromatography (eluted with chloroform/ethanol 100:3) to yield a pale yellow solid (80%), which was designated as Mn^III^PC. Mn^III^PC needs to be kept out of light at 4°C. ^1^H NMR (CDCl_3_) *δ*: 7.61 (s, 1 H), 4.85 (s, 2 H), 2.22 (t, 2 H), 1.66 (q, 2 H), 1.07-1.09 (t, 3 H).

### The Synthesis of HA-HDA

This chemical reaction is designed according to previous literature (Kaczmarek et al., 2018; Wang et al., 2020a). HA (300 mg) was dissolved in pure water (20 ml); afterward, 20 ml DMSO was added to it and the solution was stirred for 10 min. EDC⋅HCl (30.6 mg dissolved in DMSO) and NHS (18.4 mg dissolved in pure water) were added into the above solution of HA and stirred for 1 h at room temperature. Then HDA (19.3 mg dissolved in DMSO with the help of ultrasound) was dropped into the reaction dropwise. The reaction was processed for 24 h at room temperature and dialysis (MW cutoff 3.5 kDa) against water for 2 days. HA-HDA sponge was fabricated by freeze-drying.

### Preparation of the Nanoparticles

Mn^III^PC (1.0 mg), DTX (1.0 mg), and PLGA (30.0 mg) were dissolved in DCM. Then, under ultrasound conditions, the mixture above was added into the HA-HDA solution (2 mg/ml, 2 ml); the mixture was treated under the ultrasonic probe for 3 min (50%, 3 s on/3 s off). Then, the as-synthesized HA-HDA (0.5 mg/ml, 5 ml) was dropwise added into the beaker and stirred for 4 h at room temperature. The product was used to wash several times to remove the organic solvent and collected by centrifugation (12,000 rpm, 5 min). Ultimately, the nanoparticles were freeze-dried and stored in the dark at 4°C. Homogeneously, Mn^III^PC@PLGA@HA and DTX@PLGA@HA were prepared by altering the amount of Mn^III^PC and DTX (1.5 mg), respectively.

15 mg MDPH and MnCl_2_ (0.1 mol/l, 8 ml) aqueous solution were stirred at room temperature for 25 h. Then, the sample was collected by washing and centrifuging with distilled water for 3 times to obtain MDPMH. Finally, it was redissolved in 5% lactose solution, freeze-dried, and stored at −20°C.

### The Characterization of the Nanoparticles

Transmission electron microscopy (TEM) images were acquired using the Hitachi H-7650 electron microscope. Dynamic light scattering (DLS) was performed on a Malvern Zetasizer Pro (Mastersizer 3000) to determine the hydrodynamic size and zeta potential. The optical properties of nanoparticles were recorded by ultraviolet-visible spectroscopy (UV-Vis, UV-2600) from 200 to 900 nm. The extinction coefficient of MDPMH was investigated with different concentrations (0.05 × 10^−4^, 0.1 × 10^−4^, 0.2 × 10^−4^, 0.4 × 10^−4^, and 0.8 × 10^−4^ mol l^−1^).

### Drug Loading and Release Behaviors

For the determination of drug loading content (DLC) and drug loading efficiency (DLE), Mn^III^PC@PLGA@HA, DTX@PLGA@HA and MDPMH were dissolved in DMSO at a certain concentrations, respectively. Mn^III^PC was detected by high-performance liquid chromatography (HPLC, Sepiatec SEPBOX 2D-2000), while DTX was examined by UV-Vis. DLC and DLE were calculated according to the following formula:

DLC(wt.%)=(weight of loaded drug/total weight of loaded drug and carrier)×100%

DLE(%)=(weight of loaded drug/weight of the drug added)×100%

In order to investigate the drug release behavior, 1 mg MDPMH was dissolved in 1 ml phosphate-buffered saline (PBS) and transferred into a dialysis bag (MW cutoff 3.5 kDa). Then, the dialysis bag was subsequently placed in a centrifuge tube containing 30 ml PBS with different conditions, pH = 7.4, pH = 7.4 + HAase (1,000 units/ml) and pH = 5.5 + HAase (1,000 units/ml), and shaken in a water bath thermostat oscillator (SHZ-C, Shanghai) with the speed of 100 rpm at 37°C. At each specified point, 3 ml dialysate was fetched and replenished with an equal volume of fresh medium. Then, the release rates of DTX and Mn^2+^ were measured by HPLC and inductively coupled plasma-optical emission spectrometry (ICP-OES, PerkinElmer Optima 5,300 DV), respectively.

### The Photothermal Properties of MDPMH

The photothermal properties of MDPMH were studied in this section. In detail, MDPMH was dispersed in PBS solution for different concentrations (500, 1,000 and 2,000 μg/ml), which was placed in a 3-ml centrifuge tube under an 808-nm laser (2 W/cm^2^) for 10 min, respectively. The temperature was recorded by a thermocouple thermometer (TASI-605) at every minute. Similarly, the properties of MDPMH were tested under different powers of the laser (1 and 2 W/cm^2^). In addition, the thermal cycling stability of MDPMH was repeated four times. During the experiment, PBS was set as blank control. The distance between the laser source and the liquid surface was maintained at 25 mm during the irradiation.

### Stability Test

To test the stability of the drug-loaded nanoparticles, 10 μM MDPMH was dispersed in the mixture solution of DMSO and H_2_O (volume ratio = 1:10, pH = 7.4). The size and absorption properties were measured and recorded by DLS and UV-Vis every day, respectively.

### CCK-8 Assay

A549 cells were seeded in a 96-well plate at a density of 5 × 10^3^ cells per well overnight in an incubator (37°C, 5% CO_2_). Then, cells were co-incubated with DTX@PLGA@HA, PLGA@HA, MDPMH, Mn^III^PC@PLGA@HA, Mn^III^PC@PLGA@HA + Laser, and MDPMH + Laser at different concentrations (500, 1,000 and 2,000 μg/ml), respectively. In addition, the PBS and laser groups were used as control groups. The photothermal group was irradiated by near-infrared laser (808 nm, 2 W/cm^2^) for 4 min each well, and each group was cultured for another 4 h. Then, the solution was discarded and washed with PBS. A 100-μl solution containing 10% CCK-8 was added and incubated for 2 h. The microplate reader (Bio-Rad, Hercules, CA, USA) was used to measure and record the absorbance (OD value) at the 450-nm wavelength. The survival rate was acquired by the following formula:

Survival rate (%) = (experimental hole OD value-blank hole OD value)/(control group OD value-blank hole OD value) × 100%

### Cell Apoptosis

Annexin V-FITC/PI staining assay was performed to measure the A549 cell apoptosis after different treatments. The A549 cells were seeded in the 24-well plate at the density of 2 × 10^5^ cells per well and incubated for 12 h. The cells were treated with different treatments (PBS, Laser, PLGA@HA, DTX@PLGA@HA, Mn^III^PC@PLGA@HA, Mn^III^PC@PLGA@HA + Laser, MDPMH, and MDPMH + Laser) for 24 h. The cells were collected and washed twice by PBS. The apoptosis rate was determined by Annexin V-FITC/PI Kit and flow cytometric assay.

### Calcein AM/PI Dual Staining

The cytotoxicity of MDPMH nanoparticles *in vitro* was further investigated by calcein AM/propidium iodide (PI) dual-staining methods. Briefly, A549 cells (5,000 cells per well) were incubated in a 96-well plate for 24 h. Then, each group with the same concentration (2,000 μg/ml) conducted the following steps as CCK-8. Subsequently, the above cells were dyed by Calcine AM (2 μM) and PI (4.5 μM) at 37°C for 15 min. Finally, fluorescence images were taken under a laser scanning confocal microscopy (TCS-SP8, Leica, Germany).

### Western Blot Analysis

A549 cells were inoculated 2 × 10^5^ cells per well in a 6-well plate and cultured overnight (37°C, 5% CO_2_). Then, PBS (control group), MnCl_2_ (50 μM), MDPMH, and MDPMH + Laser were added to each group in sequence. Only the MDPMH + Laser group was irradiated by a near-infrared laser (808 nm, 2 W/cm^2^) for 4 min, and each group was cultured for 12 h. Then, cells were harvested and treated according to the manufacturer’s instructions. The protein concentrations were detected by the BCA kit. Protein samples were separated on SDS-PAGE gels and transferred to polyvinylidene difluoride membranes. The membranes were blocked with 5% BSA and incubated with anti-beta actin (1:1,000, ab8226), anti-cGAS antibody (1:1,000, ab224144), anti-Bax antibody (1:1,000, ab32503), and anti-Bcl-2 antibody (1:1,000, ab32124), respectively. Then the relevant horseradish peroxidase-conjugated secondary antibodies were inculcated. Ultimately, proteins were detected using the chemiluminescent detection reagents. Besides, the expression of cGAS with different concentrations of MnCl_2_ was also evaluated.

### Statistical Analysis

Results were expressed as mean ± SD. Data were analyzed by *t*-test with the scientific statistic software GraphPad Prism 7.00.

## Results

### The Preparation and Characterization of the Nanoparticles

**FIGURE 2:**
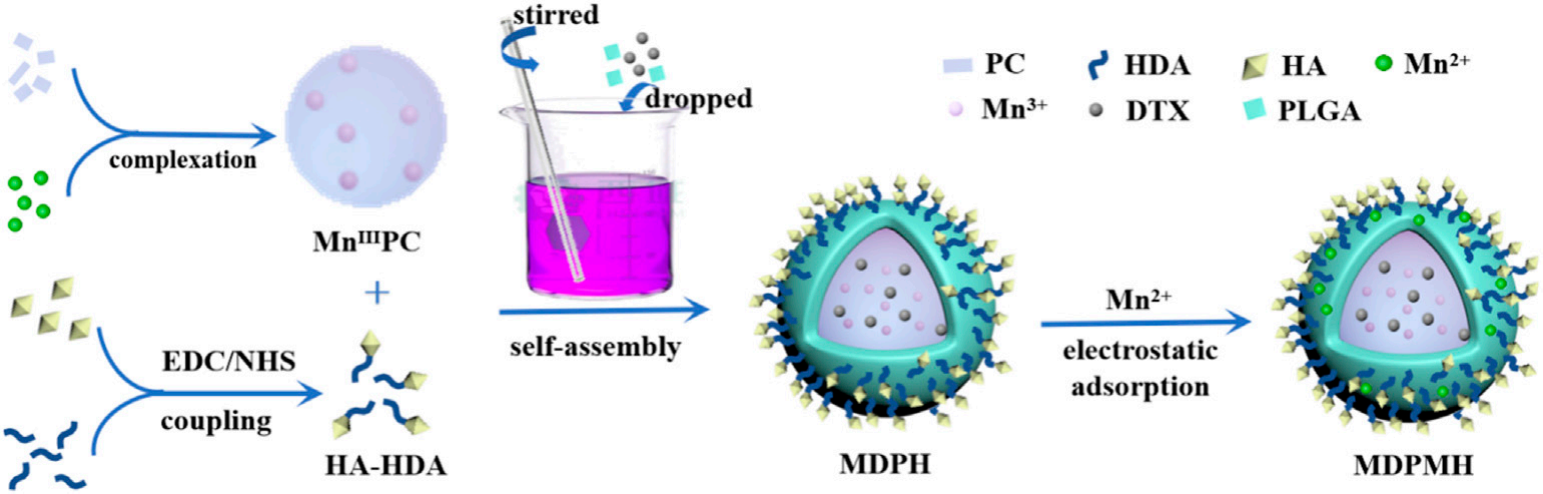
The synthetic route of MDPMH.

Generally, the synthesis route of the MDPMH nanoparticles was described as follows (Figure 2). Firstly, the novel photothermal agent Mn^III^PC was synthesized by (BuO)_8_PCH_2_ and MnCl_2_. HA and HDA were coupled by the catalysis of the EDC·HCl and NHS. Secondly, MDPH was acquired by the mixture of HA-HDA, Mn^III^PC, DTX, and PLGA. Finally, the “one-for-all” nanoplatform—MDPMH—was obtained by the electrostatic absorption between MDPH and Mn^2+^. The spectra of ^1^H NMR and mass spectrometry are shown in Supplementary Figure S1A,B, respectively. The ^1^H NMR spectrum exhibited in Supplementary Figure S1C demonstrated the successful synthesis of PLGA@HA modified by HDA.

**FIGURE 3:**
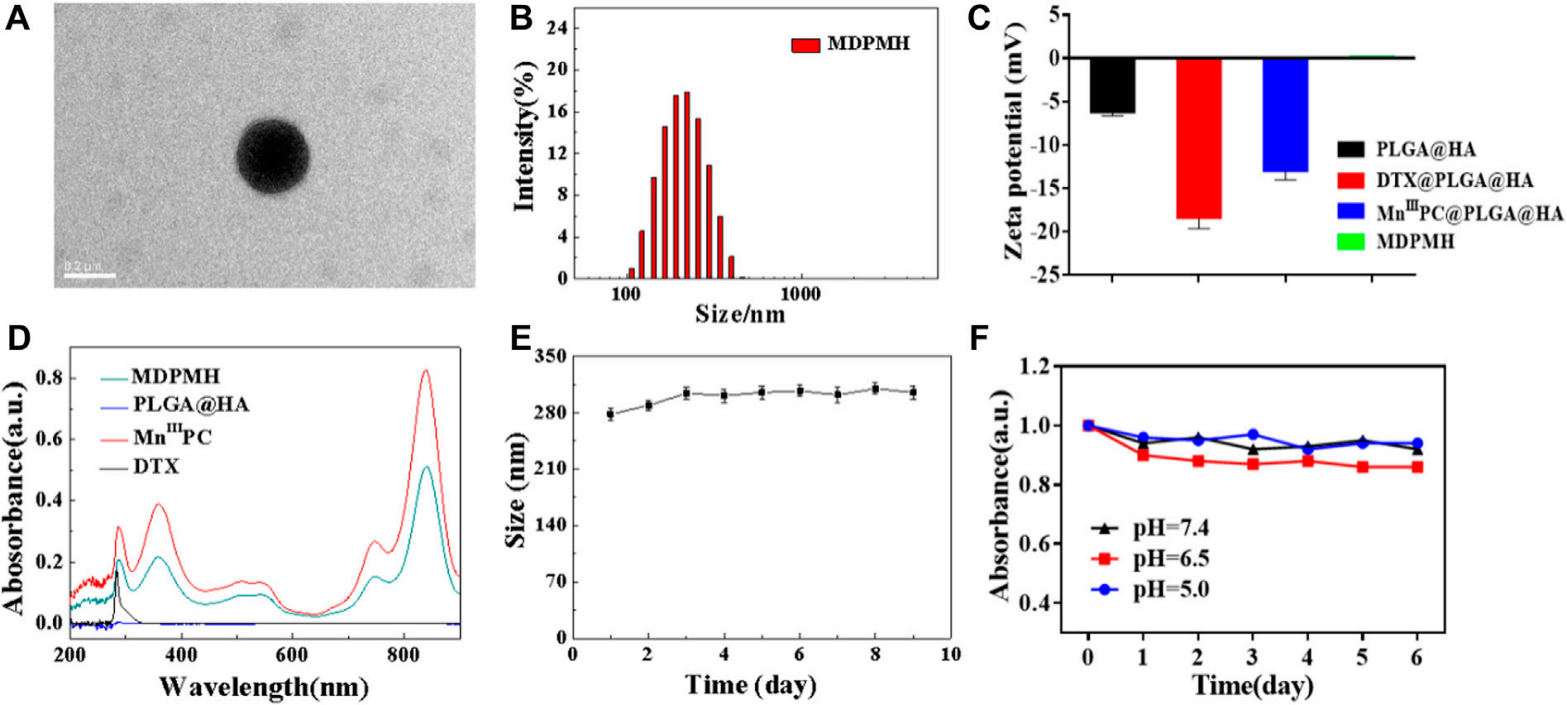
**(A)** TEM image MDPMH, scale bar = 0.2 μm. **(B)** The size distribution of MDPMH. **(C)** Zeta potential of PLGA@HA, DTX@PLGA@HA, Mn^III^PC@PLGA@HA, and MDPMH. **(D)** The UV-vis spectra of DTX, Mn^III^PC, PLGA@HA, and MDPMH from 200 to 1,000 nm. **(E)** The size change of the MDPMH dispersed in the mixture solution of DMSO and H_2_O. **(F)** The absorption spectrum of MDPMH nanoparticle under different pH conditions (5.0, 6.5, 7.4) in 6 days.

MDPMH nanoparticles were measured by TEM with a diameter of about 210 nm (Figure 3A). The size of the nanoparticles was further evaluated using DLS, which was mainly distributed around 230 nm (Figure 3B). The reason for the difference was possibly owing to the physical state of samples. Then, the zeta potential of each nanoparticle (PLGA, DTX@PLGA@HA, Mn^III^PC@PLGA@HA, and MDPMH) was determined by -6.18, -18.4, -13.0, and 0.143 mV, respectively (Figure 3C). According to the previous reports, the negatively charged or slightly positively charged nanoparticles had better blood circulation stability (Poon et al., 2011). Furthermore, slightly positively charged nanoparticles are beneficial to improving the uptake efficiency of tumor cells. The UV-Vis absorbance spectra of the several samples were measured from 200 to 900 nm and are shown in Figure 3D. There were no obvious peaks in PLGA@HA. Particularly, the absorbance at 282 and 832 nm was detected, indicating the absorption of the B band and Q band from the π electron transition on the benzene ring in the structure of phthalocyanine (Berríos et al., 2007; Göksel, 2016). The UV-vis absorbance spectra demonstrated the successful synthesis of MDPMH. The extinction coefficient of MDPMH was studied by detecting the absorbance at 832 nm with the different concentrations of the sample (Supplementary Figure S2), which were 1.025×10^4^ l mol^−1^·cm^−1^. Additionally, the DLS and UV-Vis results demonstrated the good stability of MDPMH (Figures 3E,F).

### Drug Loading and Release Behaviors

The DLC and drug loading efficiency (DLE) of different nanoparticles were detected and are shown in Table.1. The standard curve of DTX is shown in the supporting information. The DLC of Mn^III^PC in Mn^III^PC@PLGA@HA was 11.58%, while Mn^III^PC in MDPMH was 3.15%, which might be attributed to the higher total mass of nanoparticles; The DLC of DTX in DTX@PLGA@HA was 28.07%, while the DTX in MDPMH was 37.46%, indicating the stronger absorption effect of DTX. The DLE of Mn^III^PC in Mn^III^PC@PLGA@HA and DTX in DTX@PLGA@HA was 78.00% and 87.00%, respectively. Additionally, the DLE of Mn^III^PC and DTX reached 54% and 64%, respectively. The DLC of DTX presented an increasing trend after several drug-loading procedures, which was beneficial to the preparation of the nanoparticles. These results demonstrated that the MDPMH nanocarrier system had a higher loading capacity of Mn^III^PC and DTX.

**TABLE 1:** The drug loading content (DLC) and drug loading efficiency (DLE) in different nanoparticles.

	DLC (wt%)	DLE (%)
Mn^III^PC@PLGA@HA	11.58%	78.00%
DTX@PLGA@HA	28.07%	87.00%
MDPMH	Mn^III^PC: 3.15%:DTX: 37.46%	Mn^III^PC: 54%; DTX: 64%

**FIGURE 4:**
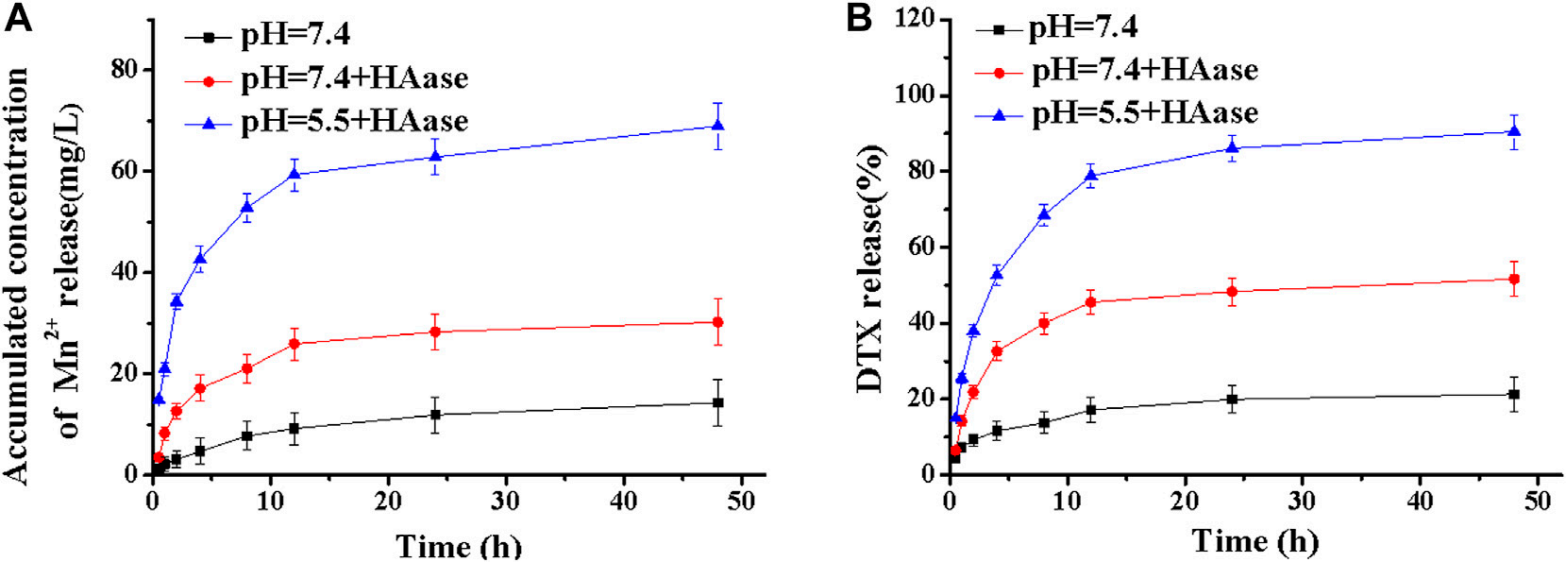
Mass of cumulative Mn^2+^ **(A)** and mass percentage of DTX **(B)** released from MDPMH in buffer solutions (pH = 7.4, pH = 5.5 + HAase and pH = 7.4 + HAase) at 37°C.

To evaluate the release performance of the MDPMH nanoplatform, the *in vitro* release of Mn^2+^ and DTX was investigated within 48 h (Figure 4A). As shown in Figures 4A,B, the concentration of Mn^2+^ and the release rate of DTX were not obvious while the Mn^2+^ concentration of pH = 5.5 + HAase exhibited the highest, which mainly owed to the degradation of HA on the surface of MDPMH under the stimulation of HAase, and the weakly acidic conditions further promoted the release of Mn^2+^ from MDPMH. Similarly, the release rate of DTX in Figure 4B also followed the regularity. The above experiments showed that MDPMH had good enzyme response release performance.

**FIGURE 5:**
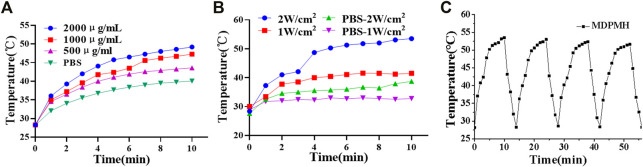
Photothermal curves of the MDPMH at the various conditions with an 808-nm laser, PBS as the control group. **(A)** The photothermal effect of concentrations (500, 1,000 and 2,000 μg/ml) at the power energy of 1 W/cm^2^. **(B)** The effect of laser power (1 W/cm^2^, 2 W/cm^2^). **(C)** The temperature variations of MDPMH at 2,000 μg/ml under 808-nm laser irradiation for 4 cycles.

The photothermal properties of the MDPMH NPs were evaluated under 808-nm near-infrared laser irradiation (Figure 5). Phosphate buffer solution (PBS) containing the various concentrations of MDPMH Nanoparticles (500, 1,000 and 2,000 μg/ml) was irradiated with an 808-nm (2 W/cm^2^) laser for 15 min, and the temperature was monitored. As shown in Figure 5A, the solution temperature increased with the increasing concentration of MDPMH, indicating a concentration-dependent photothermal effect. Then, the effects of laser Figure 5B power were examined (Figure 5B). The temperature of MDPMH solution could reach 53.5°C while the laser power was 2 W. Intriguingly, MDPMH nanoparticles could still reach 51.6°C after 808-nm laser irradiation for 4 cycles (Figure 5C). The results manifested that MDPMH has good photothermal conversion efficiency and photothermal stability.

### Cell Viability and Cytotoxicity Performance *in vitro*

**FIGURE 6:**
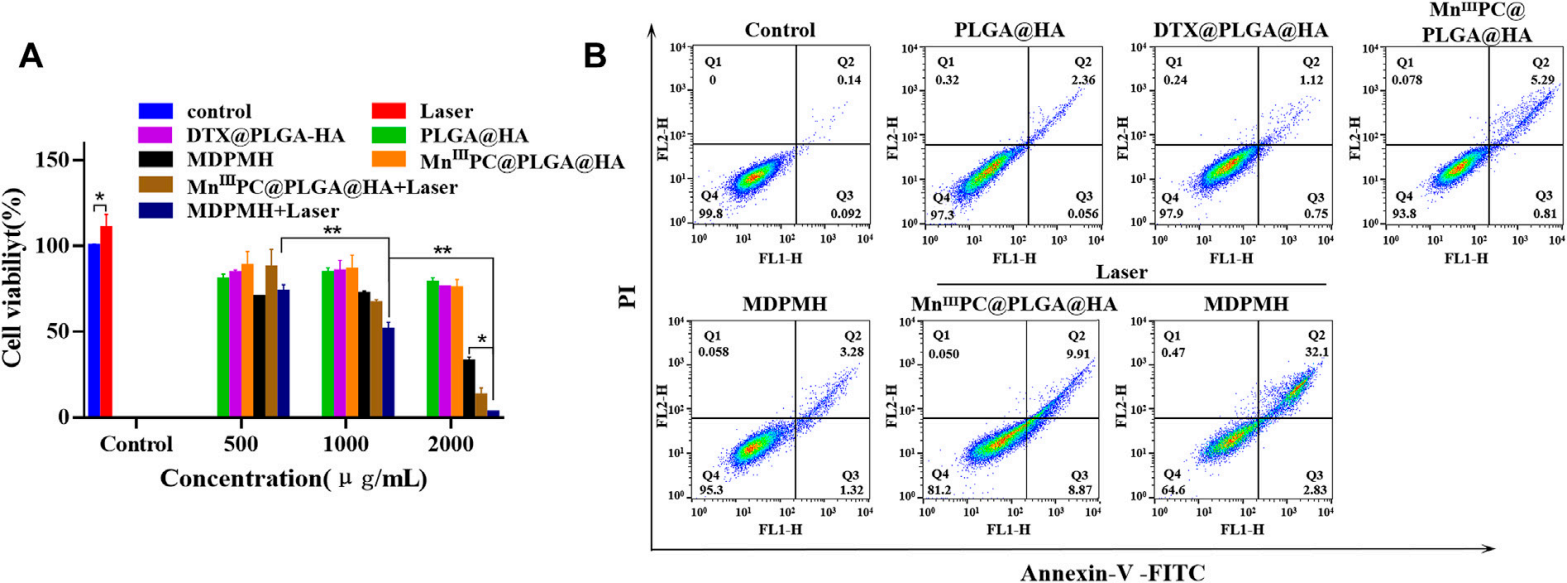
**(A)** The cytocompatibility assessment of different nanoparticles on A549 by CCK-8 assay. **(B)** Flow cytometric analysis on the apoptosis levels of A549 cell incubation with Control, PLGA@HA, DTX@PLGA@HA, Mn^III^PC@PLGA@HA, MDPMH, Mn^III^PC@PLGA@HA + Laser, and MDPMH + Laser, *p < 0.05, **p < 0.01.

The cell viability of MDPMH *in vitro* was investigated by CCK-8 assay. Different groups [PBS (Control), Laser, PLGA@HA, Mn^III^PC@PLGA@HA, Mn^III^PC@PLGA@HA + laser, DTX@PLGA@HA, MDPMH, and MDPMH + laser] and concentrations (500, 1,000, 2,000 μg/ml) were studied on the growth of the A549 cells (Figure 6A). Compared with the control group, the cell activity of the Laser group did not change significantly. With the increase in the concentration, the cell activity of the PLGA@HA group did not decrease significantly, indicating that the nanocarrier is not the cause of A549 cytotoxicity. Under an 808-nm laser, MDPMH exhibited the lowest viability (around 5%) when the concentration was up to 2,000 μg/ml. The group of PLGA@HA showed little damage to the A549 cells. Although there was some injury to A549 cells from the groups of Mn^III^PC@PLGA@HA nanoparticles with laser and DTX@PLGA@HA, the cell survival rate was significantly lower than MDPMH with 808-nm irradiation. These results demonstrated that the obtained nanosystem could effectively inhibit the proliferation of tumor cells with the help of irradiation. Moreover, the antitumor effects from the apoptosis assays of various treatment groups were evaluated. As shown in Figure 6B, the PBS, PLGA@HA, DTX@PLGA@HA, Mn^III^PC@PLGA@HA, and MDPMH groups barely affected the apoptosis of A549 cells, while Mn^III^PC@PLGA@HA with light irradiation (808 nm, 2 W/cm^2^) and MDPMH could promote the apoptosis of tumor cells, whose apoptosis ratio was ∼20% and ∼40%, respectively. As expected, the apoptosis ratio of cells treated by MDPMH under laser irradiation was higher than that of other groups, which could probably be attributed to the effect of PTT. Previous studies had confirmed that PTT could further enhance the permeability of the tumor cell membrane and thus improve the internalization of nanoparticles (Liu et al., 2019; Huang et al., 2021).

**FIGURE 7:**
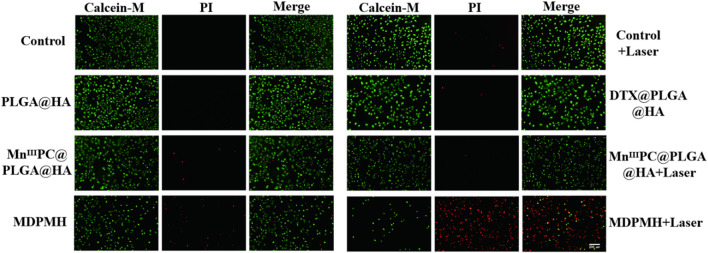
A549 cells stained with calcein-AM under different conditions; live imaging was observed by a fluorescence microscope. Scale bars = 50 µm.

To further confirm the cytotoxicity of MDPMH nanoparticle *in vitro*, different nanoparticles were investigated by Calcein-AM/PI staining, respectively (Figure 7). Significantly, the strong green fluorescence appeared in the Control, Laser, and PLGA@HA groups, which showed good biocompatibility of nanocarriers and lasers. Compared with the DTX@PLGA@HA and Mn^III^PC@PLGA@HA groups, the green fluorescence intensity weakened in the MDPMH group. Intriguingly, the MDPMH + Laser group has the strongest red fluorescence and the weakest green fluorescence, indicating the lowest cell viability with the irradiation of laser. Those results demonstrated that MDPMH might have synergistic antitumor effects of photothermal, chemotherapy, and immunotherapy.

### Western Blot

**FIGURE 8:**
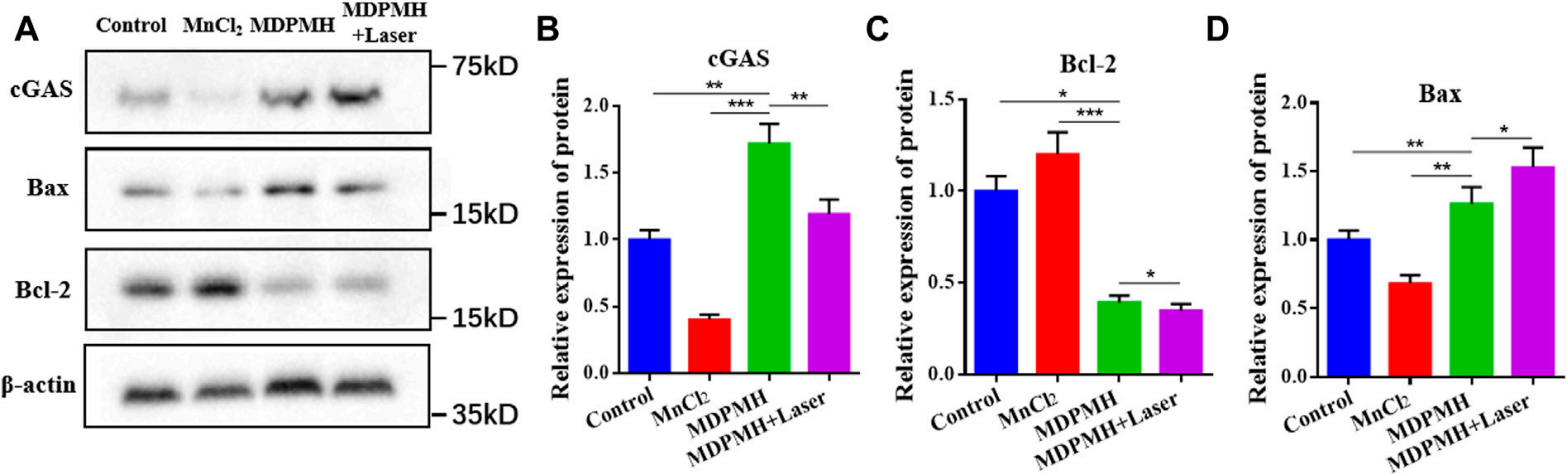
**(A)** Western blot analysis on the expression of cGAS, Bax, and Bcl-2 in A549 cells after incubation with Control, MnCl_2_, MDPMH, and MDPMH + Laser; β-actin was selected as internal reference protein. The relative expression of different proteins, cGAS **(B)**, Bax **(C)**, and Bcl-2 **(D)**. *p < 0.05, **p < 0.01, ***p < 0.001.

To verify whether the release of Mn^2+^ could activate tumor immunity, the protein expression changes of cGAS, Bax, and Bcl-2 were detected with β-actin as internal reference protein. The corresponding results are shown in Figure 8. From Figure 8A, the expression of Bcl-2 was reduced after medication, while the expression of Bax was higher, which represents the increased apoptosis corresponding with the references (Hassan et al., 2014; Zhang et al., 2019). The corresponding gray values are shown in Figures 8B–D, which demonstrated the quantitative changes in relative protein expression. Additionally, the cGAS band in the MnCl_2_ and control groups was shallow. It might be that the short retention time and low concentration of Mn^2+^ and the cGAS-STING pathway cannot be effectively activated. Supplementary Figure S4 further clarified the relationship between the concentration of Mn^2+^ and the expression of cGAS. The group of MDPMH and MDPMH + Laser exhibited more deeply, which could probably be attributed to the Mn^2+^ released from MDPMH which could trigger the tumor immune response in the tumor by activating the cGAS-STING pathway (Hopfner and Hornung, 2020; Hou et al., 2020; Wang et al., 2020b). The proteins Bax and Bcl-2 are associated with cell apoptosis. Preclinical studies have shown that tumor PTT is associated with host-specific antitumor immune response (Zhang et al., 2020). After tumor hyperthermia, the immune function of the host body changes, and the antitumor immune response of the body is activated (Xu and Liang, 2020).

## Discussion

In this paper, a nanoplatform composed of HA, Mn^III^PC, DTX, etc., was prepared for the NSCLC through PTT, chemotherapy, and immune therapy combination. The UV-visible spectra demonstrated the successful preparation of the nanoparticles (Rajawat and Malik, 2020). The MDPMH nanoparticles were obtained by easy methods such as self-assembly and coagulation, with uniform size distribution, spherical shape, and good stability. Under the 808-nm laser irradiation, MDPMH showed the optimal ability to clear the tumor cells by increasing the expression of apoptosis-related proteins and reducing the expression of proteins that inhibit apoptosis because of the loading of DTX and Mn^III^PC. Besides, once Mn^2+^ could be released from MDPMH, it would activate the immune function of host body changes and the systemic antitumor immune response of the host, which could help remove tumor cells. However, the nanodelivery platform still needs to be improved. An example is the DLC. All in a word, the above mechanisms promise a localized drug-delivery platform for enhancing the therapeutic efficacy of PTT, chemotherapy, and immune therapy combination.

## Conclusion

In summary, a smart nanodelivery platform was designed which combined multimodal therapy. The synthesized MDPMH with slightly positive electrical properties and surface-modified HA was beneficial to the uptake of NSCLC cells. Under 808-nm laser irradiation, the nanoplatform exhibited good PTT effect and photothermal stability. Importantly, MDPMH could effectively inhibit the growth of A549 cells, which was mainly ascribed to the coordinated effects of DTX, Mn^III^PC, and Mn^2+^ in the nanocarrier system. The combined strategy may exploit great therapeutic effects and development potential.

## Data Availability

The original contributions presented in the study are included in the article/Supplementary Material, further inquiries can be directed to the corresponding author.
