# Corrigendum: Docetaxel-loaded novel nano-platform for synergistic therapy of non-small cell lung cancer

**DOI:** 10.3389/fphar.2022.964540

**Published:** 2022-09-07

**Authors:** Xing Feng, Xiaoling Xiong, Shenglin Ma

**Affiliations:** ^1^ Department of Thoracic Surgery, The Affiliated Hangzhou Hospital of Nanjing Medical University, Hangzhou, China; ^2^ Department of Thoracic Surgery, Affiliated Hangzhou First People’s Hospital, Zhejiang University School of Medicine, Hangzhou, China; ^3^ Department of Nephrology, Sir Run Run Shaw Hospital, Zhejiang University School of Medicine, Hangzhou, China; ^4^ Department of Oncology, The Affiliated Hangzhou Hospital of Nanjing Medical University, Hangzhou, China

**Keywords:** non-small cell lung cancer, targeted therapy, immunotherapy, photothermal therapy, cGAS-STING

In the published article, there was an error in the author list. Author Xing Feng was erroneously listed as corresponding author. The corrected author list appears below:

“Xing Feng^1,2^, Xiaoling Xiong^3^ and Shenglin Ma^4^*

*Correspondence: Shenglin Ma, mashenglin0607@163.com”

The authors apologize for this error and state that this does not change the scientific conclusions of the article in any way. The original article has been updated.

