# Supplementary material for: Docetaxel-Loaded Novel Nano-Platform for Synergistic Therapy of Non-Small Cell Lung Cancer
Source: Front Pharmacol. 2022 Mar 2;13:832725. doi: 10.3389/fphar.2022.832725 (PMC8926142; doi:10.3389/fphar.2022.832725)
Supplement: Supplementary file 1 [file DataSheet1.docx]

**Supporting Information**

**(a)**


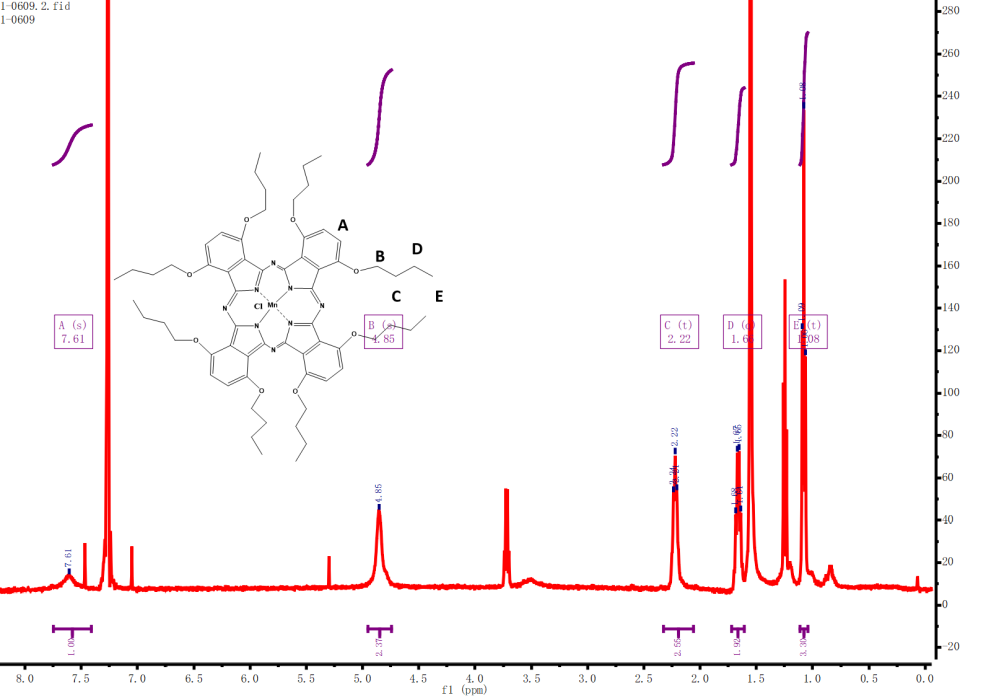


**(b)**

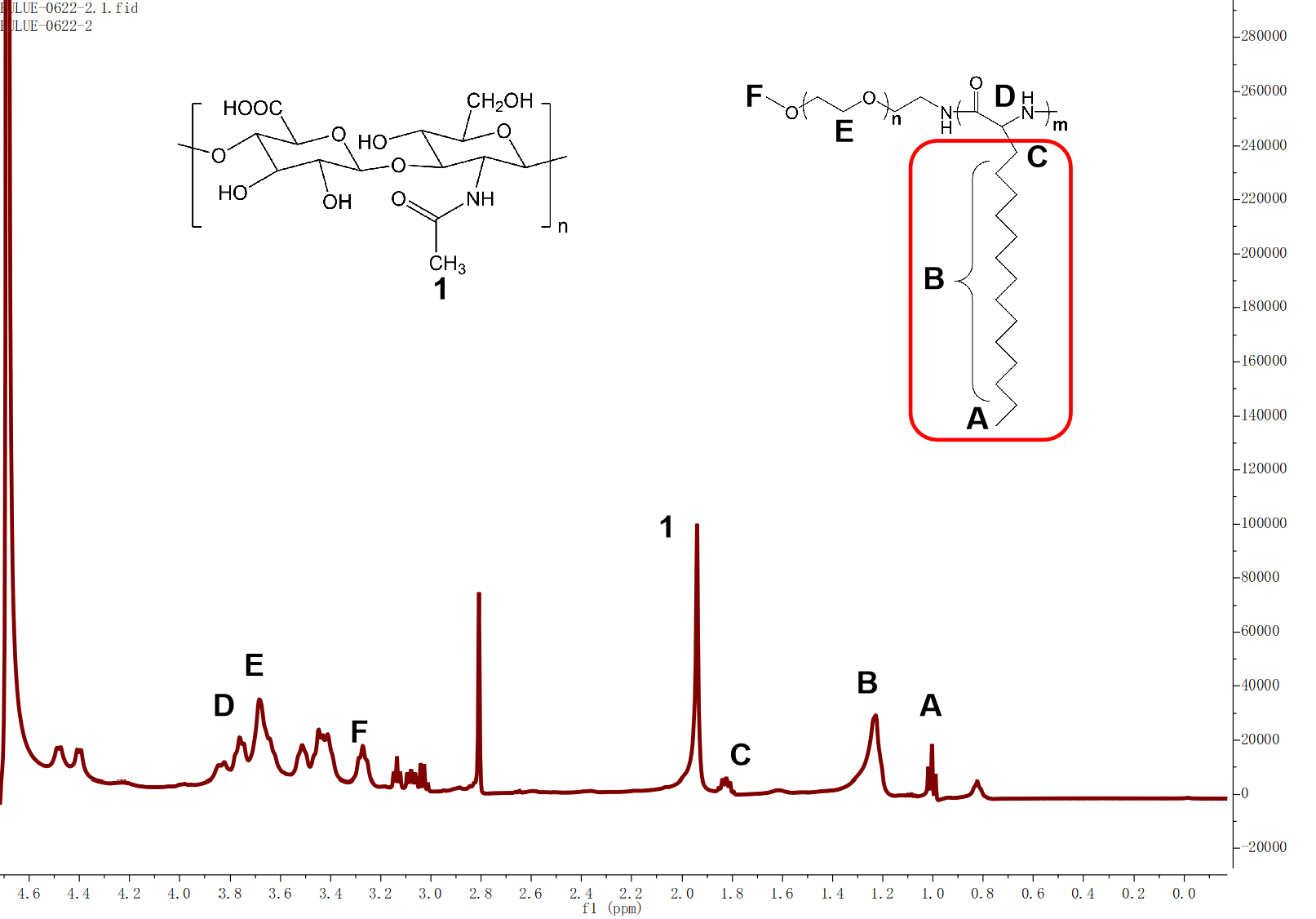


**(c)**

**Fig.S1 The 1H NMR spectrum (a) and mass spectrometry spectrum (b) of Mn^III^PC. The 1H NMR spectrum (c) of HA@PLGA modified by HDA.**

**Fig. S2 Absorbance of MDPMH solutions with different concentrations (n=3)**

**Fig. S3 The standard curve of DTX detected by HPLC**


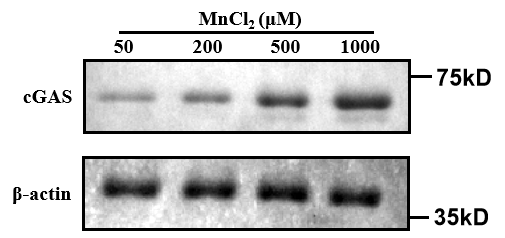


**Fig. S4 Western blot assay on the expression of cGAS with different concentration of MnCl_2_**
